# Predicting Hypertensive Disease in the First Trimester of Pregnancy: Risk Models and Analysis of Serum D-dimer Levels Combined with Plasma Pregnancy-Associated Protein A, Free *β*-Subunit of Human Chorionic Gonadotropin, and Fetal Nuchal Translucency

**DOI:** 10.1155/2022/8264958

**Published:** 2022-03-30

**Authors:** Yiming Chen, Wenwen Ning, Xuelian Chu, Yijie Chen, Linyuan Gu, Zhen Xie, Liyao Li, Caihe Wen, Xiaoying Wang

**Affiliations:** ^1^Department of Prenatal Diagnosis and Screening Center, Hangzhou Women's Hospital (Hangzhou Maternity and Child Health Care Hospital), Hangzhou, Zhejiang 310008, China; ^2^Department of the Fourth School of Clinical Medical, Zhejiang Chinese Medical University, Hangzhou, Zhejiang 310053, China; ^3^Department of Laboratory, Maternal and Child Health Hospital of Yuhang District, Hangzhou, Zhejiang 311100, China; ^4^Department of Obstetrics, Hangzhou Women's Hospital (Hangzhou Maternity and Child Health Care Hospital), Hangzhou, Zhejiang 310008, China

## Abstract

We aimed to investigate the predictive ability of serum levels of D-dimer (DD) in the first trimester for the occurrence of hypertensive disorders of pregnancy (HDP). In this retrospective, case-cohort study, we measured the levels of DD, plasma pregnancy-associated protein A (PAPP-A), and free *β*-subunit of human chorionic gonadotropin (free *β*-hCG) and analyzed fetal nuchal translucency (NT) in 150 healthy gravidas, 126 cases of gestational hypertension (GH), 53 cases of preeclampsia (PE), and 41 cases with severe preeclampsia (SPE). Likelihood ratio models and risk models were built using single markers (DD, PAPP-A, free *β*-hCG, and NT) and combinations of those markers. Analyses showed that the levels of DD multiple of the median (MoM) in the GH, PE, and SPE groups were all significantly higher than those in the control group, with significant differences between groups (*χ*^2^ = 70.325, *P* < 0.001). The area under curve (AUCs) for DD in the GH, PE, and SPE groups was 0.699, 0.784, and 0.893, respectively; the positive likelihood ratio (+LR) was 1.534, 1.804, and 2.941, respectively; and the negative likelihood ratio (-LR) was 0.022, 0.081, and 0, respectively. When the cut-off values of DD for the GH, PE, and SPE groups were 0.725, 0.815, and 0.945 MoM, respectively, the corresponding sensitivities were 0.992, 0.962, and 1.000, respectively. As gestational hypertension progressed, the levels of DD tended to increase gradually. The maternal serum level of DD in the first trimester had correlative and diagnostic value for HDP. The sensitivity and specificity of maternal serum levels of DD level in the first trimester for different types of HDP were significantly different; the best sensitivity and specificity were detected in the SPE group. First trimester DD level, combined with other biochemical markers, may improve our ability to diagnose HDP.

## 1. Introduction

Hypertensive disorders of pregnancy (HDP) affect approximately 10% of pregnant women, and if unrecognized and untreated, can lead to adverse maternal and fetal events, including an increased risk of maternal stroke and death, reduced fetal birth weight, and an increased risk of neonatal intensive care [1]. Preeclampsia (PE), the most representative type of HDP, is the second cause of maternal death following embolization [1, 2]. However, the etiology and pathogenesis of PE has yet to be fully elucidated, thus, creating a serious threat for the health of mothers and perinatal infants. Previous studies have shown that a low-dose of soluble aspirin before 16 weeks of gestation is effective in preventing PE, and that the scope of prenatal screening should be expanded to include pregnancy and other complications. Therefore, early detection, early prevention, and early treatment, can reduce the occurrence of PE [3].

The combination screening of plasma pregnancy-associated protein A (PAPP-A), free *β*-subunit of human chorionic gonadotropin (free *β*-hCG) and fetal nuchal translucency (NT), is widely used for the prenatal screening of trisomy 21 and trisomy 18 in the first trimester (9 weeks to 13 weeks and 6 days) [4, 5]. At a false-positive rate of 5%, this screening method can detect 81% to 96% of fetal chromosomal aneuploidy [6, 7]. PAPP-A and free *β*-hCG are also valuable for predicting PE, especially when combined with clinical risk factors of PE (advanced age, history of hypertension, and BMI during pregnancy), and can also improve the success rate of predicting PE [8, 9]. However, our previous studies showed that low levels of maternal serum PAPP-A and free *β*-hCG in early pregnancy were predictive markers for HDPs such as GH and PE. Furthermore, the diagnostic value of combined screening strategies was better than that of single screening. NT had no diagnostic value for predicting GH and PE, although the combination of NT, PAPP-A, and free *β*-hCG, screening could improve the prediction efficiency for HDPs [10].

DD is the smallest fragment cleaved by fibrin under the mediation of plasminase. Furthermore, DD has traditionally been used as a laboratory marker for venous thromboembolism disease (VTE). In normal pregnancies, DD concentrations gradually increase as pregnancy progresses [11, 12]. Kim et al. suggested that maternal levels of DD in patients with severe GH were significantly higher than those in patients with mild GH [13]. Pinheiro et al. showed that increased plasma levels of DD in the third trimester were associated with PE [14]. Given that PAPP-A, free *β*-hCG, NT, and DD may be associated with HDP, several studies have attempted to predict HDPs using these indicators in the first trimester. Therefore, in this study, we detected the serum levels of PAPP-A, free *β*-hCG, DD, and fetal NT in the first trimester of pregnancy in 126 patients with GH,53 patients with PE, 41 patients with SPE, and 150 women with normal pregnancies during the same period. Then, we compared the levels of these indicators across different subgroups and evaluated the diagnostic value of single factors, and a combination of factors for HDP.

## 2. Materials and Methods

### 2.1. Study Participants

This was a retrospective and case-control study in which we collected case data from the electronic medical records system of Hangzhou Women's Hospital (Hangzhou Maternity and Child Health Care Hospital). We collected data from 29,096 pregnant women who visited the Obstetrics Department between November 2014 and April 2019. After eliminating repeated test results, 370 pregnant women met our study requirements and were randomly selected from a sample database containing serum samples, including 150 normal pregnant women (spontaneous, live birth, and without other complications), 126 cases of GHP, 53 cases of PE, and 41 cases of SPE. This study was approved by the medical ethics committee of Hangzhou Women's Hospital [2020] Medical Ethics Review A (10)-11.

### 2.2. Diagnostic Criteria and Exclusion Criteria

The diagnostic criteria used in this study were based on the Chinese Medical Association Obstetrics and Gynecology Branch Pregnancy Hypertension Disease Group. “Guidelines for Diagnosis and Treatment of Hypertension during Pregnancy (2015)”[15]. According to the severity of disease development, HDP was divided into GH, PE, and SPE.

The exclusion criteria were as follows: (1) twin and multiple pregnancies, (2) patients who were complicated by chronic hypertension, heart disease, kidney disease, diabetes, hyperthyroidism, connective tissue disease, blood disease, and other chronic diseases, (3) smokers, (4) pregnancies arising from assisted reproductive technology, (5) trisomy 21, 18, 13 and other birth defects, (6) patients with a history of immunotherapy and blood transfusion, (7) patients with a history of special medication during pregnancy, (8) cases that were missing key information from their medical records, and (9) cases where there were mismatches between the maternal information and serum samples.

### 2.3. Reagents and Apparatus

PAPP-A and free *β*-hCG were detected using a 1235 Automatic time-resolved Fluoroimmunoassay System (PerkinElmer, USA) with matching PAPP-A, a free *β*-hCG kit, an enhancer, washing liquid, quality control samples, and a range of standards (PerkinElmer, USA). DD was detected using a Rt-6100 Plate Reader (Rayto, Shenzhen, China), 988 Plate Washer (Tianshi, Beijing), and DD kit (BIM, San Francisco, USA). The thickness of the fetal cervical hyaline layer was measured by a Voluson E8 ultrasonic system (GE, Boston, USA).

### 2.4. Subjects and Methods

#### 2.4.1. Detection of PAPP-A, Free *β*-hCG, and DD samples

At 9 to 13 weeks and 6 days of gestation, 2-3 ml of venous blood was aseptically collected from fasting pregnant women and placed into vacuum separation tubes. The tubes were immediately mixed upside down, and centrifuged at 3000 rpm for 15 min. The serum samples were separated for 30 mins and then stored in a refrigerator at 2°C-8°C and analyzed within one week. After PAPP-A and free *β*-hCG levels had been detected by the A1235 Automatic Immunoassay System (PerkinElmer, Shelton, CT, USA), the remaining serum was stored at -80°C. After accumulating a certain number of cases, information from each group was matched with the stored serum samples, and the serum samples were centrifuged and mixed at low temperature. The levels of DD were detected by enzyme-linked immunosorbent assays (ELISAs) with the double antibody one-step sandwich method.

#### 2.4.2. Detection of Fetal NT Thickness

Fetal NT thickness was screened according to the standards issued by the Fetal Medicine Foundation (https://fetalmedicine.org/education/the-11-13-weeks-scan/). Specially trained physicians performed ultrasound examinations in accordance with standardized protocols to assess the fetal NT thickness. Images were acquired such that we had a midsagittal view of the fetus in a natural posture. We then adjusted the images so that only the fetal head and upper chest were visible. We then measured the widest transparent place between the skin and the cervical soft tissue.

#### 2.4.3. Expressions of DD, PAPP-A, Free *β*-hCG, and NT

The levels of DD, PAPP-A, free *β*-hCG and NT were expressed by multiple of median (MoM) of maternal weight and maternal age. The original concentration value was replaced by MoM value, and the MoM value was calibrated by maternal weight and gestational age [10]. MoM was defined by the following formula:

(1) $$\begin{array}{c} \text{MoM}=\frac{\text{Original}\;\text{Conj}}{\text{Median}}. \end{array}$$

“Original Conj” was the original concentration of DD, PAPP-A, free *β*-hCG, and NT, and “medium” was the median of the original concentration of the corresponding indicators. In order to reduce the deviation caused by different gestational week and maternal weight, we used the median equation of gestational week and median equation of maternal weight from Hangzhou Obstetrics and Gynecology Hospital to calibrate the MoM values of various indicators.

(2) $$\begin{array}{c} \text{GA Med}=10^{\left(-108.6+35.5\times \text{GA}‐4.253\times {\text{GA}}^{2}+0.2262\times {\text{GA}}^{3}-0.004508\times {\text{GA}}^{4}\right)}. \end{array}$$

“GA” represented gestational age and” Med” represented median. The maternal weight calibration formula was similar. The MoM value was adjusted according to the median equation, and the adjusted MoM value was used in risk modeling calculation.

(3) $$\begin{array}{c} \text{Adjusted}\_\text{MoM}=\frac{\text{MoM}}{\text{GA}\_\text{Med}\times \text{maternal weight}\_\text{Med}}. \end{array}$$

#### 2.4.4. A Comparison of Prediction Efficiencies for HDP Using Different Prediction Risk Models

Using the likelihood ratio construction method, we generated risk models for DD, PAPP-A, free *β*-hCG, and NT, as single markers and then used Python software (https://www.python.org/) to create models for combined factors. The MoM values of DD, PAPP-A, free *β*-hCG, and NT, exhibited a multivariate normal distribution. According to the modeling method for the risk calculation model [16], the corresponding parameters of marker distribution were calculated, and the distribution likelihood was calculated as HDP risk.

#### 2.4.5. The Construction of Risk Prediction Models using DD, PAPP-A, Free *β*-hCG, and NT Single Markers and a Combination of Multiple Markers

The probability density function of the normal distribution was used to calculate the sample likelihood ratio. Results were then used as a risk prediction score for samples with HDP. The lifecycle-like risk value calculation method was used to construct our models [10, 16] as follows:

The prenatal age equation [17] was defined as follows:

(4) $$\begin{array}{c} {\text{risk}}_{\text{maternal age}}=0.000627+\exp^{-16.2395+0.286*\left(\text{maternal age}-0.5\right)}. \end{array}$$

In this equation, “risk_maternal age_” represented prenatal age risk value and “maternal age” represented maternal age.

Likelihood ratio was calculated as follows:

(5) $$\begin{array}{c} \text{L}{\text{R}}_{\text{multinorm}}=\frac{\text{likelihood of HDP group}}{\text{likelihood of control group}}. \end{array}$$

Finally, HDP risk value was calculated as follows:

(6) $$\begin{array}{c} {\text{risk}}_{\text{HDP}}=\frac{1}{{\text{LR}}_{\text{multinorm}}\times {\text{risk}}_{\text{maternal}\;\text{age}}}. \end{array}$$

Ten models were built separately using the same principle: model 1 (DD MoM value alone), model 2 (PAPP-A MoM value alone), model 3 (free *β*-hCG MoM value alone), model 4 (NT MoM value alone), model 5 (DD+PAPP-A), model 6 (DD+free *β*-hCG), model 7 (PAPP-A+free *β*-hCG), model 8 (DD+PAPP-A+free *β*-hCG), model 9 (PAPP-A+free *β*-hCG+NT), and model 10 (DD+PAPP-A+free *β*-hCG+NT).

#### 2.4.6. Statistical Analysis

Databases were established using Microsoft Excel 2013 software and statistical analyses were carried out using IBM SPSS Statistics version 21.0 (SPSS, USA). The one-sample Kolmogorov-Smirnov test was used to test normality. Data were skewed and presented as median and percentiles [*M* (*P*_2.5_, *P*_97.5_)]. The Kruskal-Wallis H test was used to make comparisons within groups, while the Mann-Whitney *U* test was used for comparisons between groups. We used the likelihood ratio construction method to generate risk models for DD, PAPP-A, free *β*-hCG, and NT, single markers, and Python 3.8 software (https://www.python.org/) was used to generate models for a combination of factors. Receiver operator characteristic (ROC) curves were used to evaluate the diagnostic value of plasma DD levels for PE. The boundary value for the maximum Youden index was used as the cut-off value, and *P* < 0.05 was considered to be statistically significant.

## 3. Results

### 3.1. A Comparison of Maternal Age, Gestational Age, and Maternal Weight between Groups

The maternal age of the GH, PE, and SPE groups was higher than that of the control group; however, this did not reach statistical significance (*P* = 0.299).The gestational age of the GH, PE, and SPE groups was smaller than that of the control group, although there were no significant differences (*P* = 0.283). The maternal weight of the three case groups was significantly higher than that of the control group (*P* < 0.001), as detailed in Table 1.

### 3.2. A Comparison of Serum DD, PAPP-A, Free *β*-hCG, and Fetal NT, in Each Group

**Figure 1:**
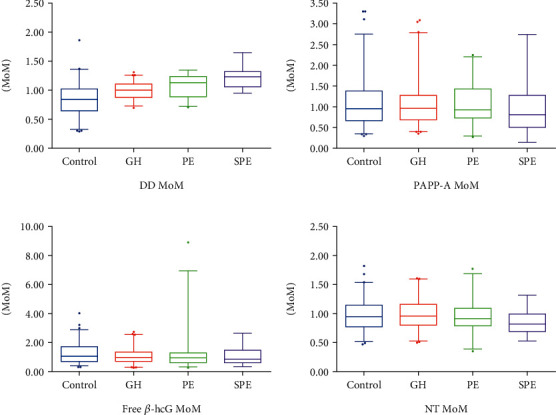
Comparison of DD, PAPP-A, free *β*-hCG, and NT MoM among the four groups. (a) DD MoM; (b) PAPP-A MoM; (c) free *β*-hCG MoM; (d) NT MoM. DD: D-dimer; PAPP-A: pregnancy-associated plasma protein A; free *β*-hCG: free beta human chorionic gonadotropin; NT: nuchal translucency; GH: gestational hypertension; PE: preeclampsia; SPE: severe preeclampsia; MoM: multiple of median.

Table 2 shows that the levels of DD in the GH, PE, and SPE groups were 1.00 (0.73-1.26), 1.13 (0.72-1.34), and 1.23 (0.95-1.64) MoM, respectively; these were higher than those in the control group (0.84 (0.33-1.36) MoM) and there were significant differences between groups (*P* < 0.001). The levels of PAPP-A and free *β*-hCG in the GH, PE, and SPE groups were lower than those in the control group, and all differences were statistically significant (all *P* < 0.001). However, the MoM values for PAPP-A and free *β*-hCG in the GH, PE, and SPE groups were lower than those in the control group; however, there were no statistically significant differences (all *P* > 0.05), as shown in Table 2. NT levels in the SPE group were all lower than those in the control group; these differences were statistically significant (*P* = 0.037). As hypertension progressed, there was a trend for maternal serum DD levels to increase progressively, as shown in Figure 1.

### 3.3. The Predictive Value of Maternal DD, PAPP-A, Free *β*-hCG, and NT alone or in combination, for GH, PE, SPE, and HDP

**Figure 2:**
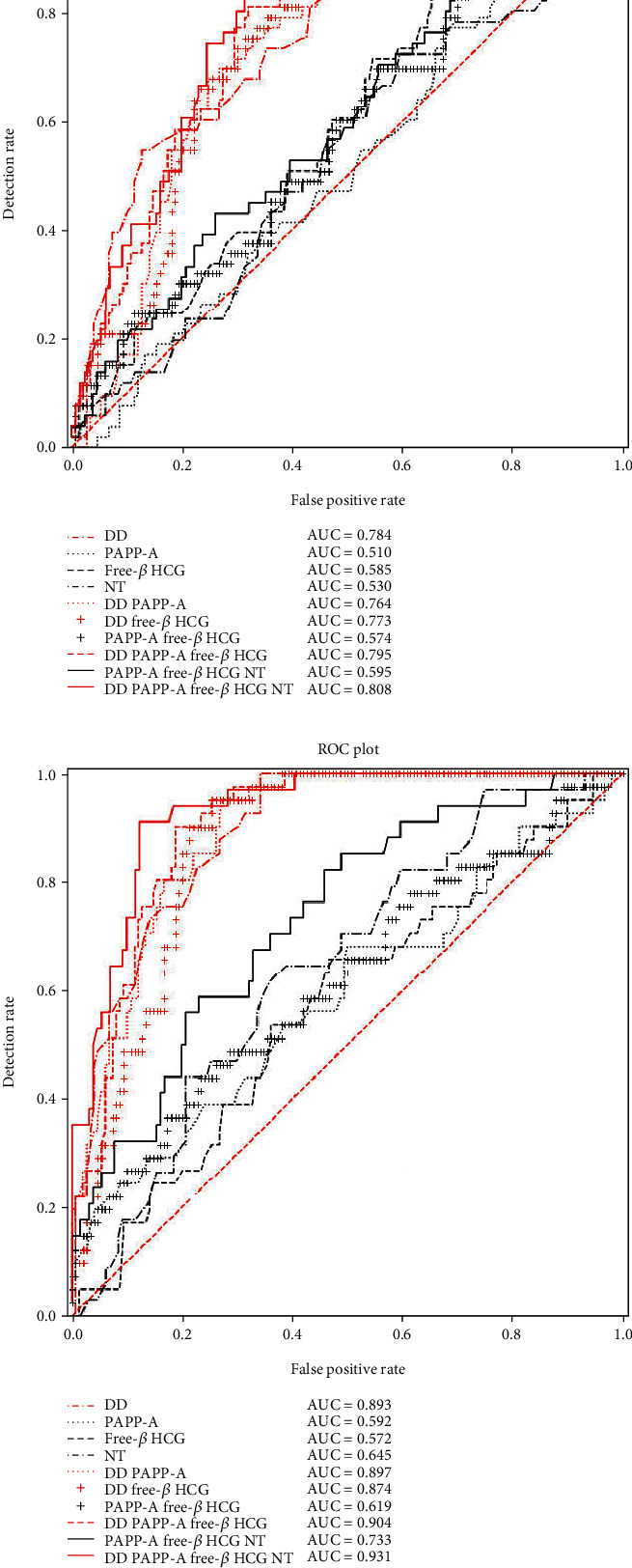
ROC curve for the prediction value of GH, PE, SPE, or HDP: (a) ROC curve for the prediction of GH; (b) ROC curve for the prediction of PE; (c) ROC curve for the prediction of SPE; (d) ROC curve for the prediction of HDP = (GH + PE + SPE). GH: gestational hypertension; PE: preeclampsia; SPE: severe preeclampsia; DD: D-dimer; PAPP-A: pregnancy-associated plasma protein A; free *β*-hCG: free beta human chorionic gonadotropin; NT: nuchal translucency; AUC: area under curve; ROC: receiver operating characteristic.

The AUCs for the GH, PE, and SPE groups with DD alone were 0.699, 0.784, and 0.893, respectively, as shown in Table 3 and Figures 2(a)–2(c). For clinical convenience, we generated ROC curves for all 220 HDP cases (from three groups: GH, PE and SPE) to predict the diagnostic value of DD for HDP with an AUC of 0.755 (0.704-0.807, *P* < 0.001). When the cut-off value of DD for HDP was 0.915 MoM, the corresponding sensitivity and specificity were 0.755 and 0.640, respectively, as shown in Figure 2(d). With regards to the diagnostic value of PE, DD+PAPP-A+free *β*-hCG+NT (AUC: 0.808) > DD+PAPP-A+free *β*-hCG (AUC: 0.795). For SPE, DD+PAPP-A+free *β*-hCG+NT (AUC: 0.931) > DD+PAPP-A+free *β*-hCG (AUC: 0.904) > DD+PAPP-A (AUC: 0.897). For HDP, DD+PAPP-A+free *β*-hCG (AUC: 0.756) > DD (AUC: 0.755), as shown in Figures 2(a)–2(d).

### 3.4. Assessment of the Value of the Risk Model

Compared with PAPP-A, free *β*-hCG and NT, the positive likelihood ratio (+LR) was highest, and the negative likelihood ratio (-LR), was lowest for DD as a single index. The +LR and -LR for DD as a single index for GH, PE, and SPE were +LR (1.534, 1.804, 2.941) and -LR (0.022, 0.081, 0), respectively. Therefore, DD as a single index had a higher predictive value than PAPP-A, free *β*-hCG, and NT, for GH, PE, and SPE, and provided the best optimal diagnostic value for SPE (+LR was highest and -LR was lowest). For multiple indices, GH was highest with +LR (2.196) for DD+PAPP-A+free *β*-hCG, while PE and SPE showed the highest +LR for DD+PAPP-A+free *β*-hCG+NT, at 2.694 and 7.465, respectively. Therefore, by adding PAPP-A, free *β*-hCG, and NT, as first trimester prenatal screening markers, the diagnostic performance improved for predicting GH, PE and SPE, as shown in Table 4.

## 4. Discussion

This was a retrospective and observational case-control study based on prenatal screening in the first trimester of pregnancy. We detected and evaluated the maternal serum levels of DD, PAPP-A, free *β*-hCG, and NT, in women with HDP (GH, PE, and SPE) and control groups. We then created a multi-index model for DD, PAPP-A, free *β*-hCG, and NT, to calculate the risk of HDP. We found that the levels of DD in the GH, PE, and SPE groups in the first trimester were significantly higher than those in the control group (*P* < 0.001). In addition, the serum levels of DD in pregnant women increased gradually with the progression of HDP, thus, suggesting that DD levels may be related to disease severity and had significant potential as a marker for predicting HDPs. Therefore, the combination of DD with other biochemical markers in early pregnancy may improve the diagnostic value of HDPs. This will allow preventive measures to be taken early to reduce the risk of diseases in pregnant women and fetuses.

Gestational hypertensive disorders affect 2-10% of pregnant women and pose significant lifetime and economic risks to pregnant woman. Women with eclampsia, chronic hypertension, and gestational hypertension, incur mean medical costs of $9,389, $6,041, and $2,237, respectively; these costs are higher than women without hypertension (*P* < 0.001) [18]. Studies have shown that women with HDP have an increased risk for almost all postpartum complications, including those that are unrelated to hypertension [19]. Furthermore, a history of HDP was found to be independently associated with poorer memory and verbal learning after 15 years of maternal pregnancy [20]. Therefore, HDPs present significant great harm to the pregnant woman; early prevention and early treatment could significantly reduce the incidence of HDPs. Therefore, exploring better predictors for HDPs has become a research hot spot in the field of perinatal medicine. In this study, we used Python 3.8 software to create 10 risk models for DD, PAPP-A, free *β*-hCG, and NT, as single and combined markers to explore the best risk model for predicting HDPs.

In normal pregnancies, the levels of DD gradually increase as pregnancy progresses, thus indicating a hypercoagulable state [13]. Our results showed that DD levels in the GH, PE, and SPE groups were higher than those in the control group; there were also statistical differences between groups (*P* < 0.001); these findings were consistent with those reported in previous studies. This study revealed a trend for progressively higher serum levels of DD in pregnant women as hypertension progressed, thus indicating that pregnant women with HDP had a more severe hypercoagulable state than normal pregnant women. Furthermore, this hypercoagulable state correlated with the severity of the disease; this finding was consistent with previous literature [21, 22]. Previous studies have found that in PE patients, this hypercoagulability continued until after delivery, and that patients with renal complications exhibited higher levels of thrombin and DD, thus, suggesting that PE may also be associated with elevated thrombin levels [22]. This suggests that the level of DD is closely related to HDP and can be used as a marker for disease severity. This also provides a research basis for the possible involvement of DD in the pathogenesis of HDP. However, our current research could not determine whether DD is a cause or a consequence of HDP; therefore, further research is needed with this regard.

With regards to the diagnostic value for SPE, DD+PAPP-A+free *β*-hCG+NT (AUC: 0.931) > DD+PAPP-A+free *β*-hCG (AUC: 0.904) > DD+PAPP-A (AUC 0.897). The risk models that involved DD generally exhibited higher AUCs than those without DD involvement; Table 4 shows that DD had the highest +LR and the lowest -LR for SPE. This indicated that prenatal screening markers in the first trimester that included DD had a better predictive effect for SPE, and that combined screening strategies had a better predictive effect for SPE. Baboulall et al. [23] previously showed that the distribution of DD was statistically different in pregnant women with SPE, and that the area under the ROC curve for DD to predict SPE was 0.828, thus, indicating the better diagnostic value of DD for SPE. In another study, Chu et al. [24] found that as HDPs worsened, DD, Fib, and vWF: Ag levels increased significantly higher in mild, moderate, and severe patients, as compared with controls. Rodríguez-Peña et al. [25] used an immunoturbidimetric assay to detect DD and found that elevated levels of DD were strongly associated with SPE. This further demonstrated that fibrinolytic activation and the activation of the coagulation system may be involved in the pathogenesis of SPE. These findings indicated that DD exhibited different diagnostic values for different types of HDP and offered the most potential for SPE.

Our research also showed that the AUCs of DD in the first trimester for GH, PE, and SPE were 0.699, 0.784 and 0.893, respectively; these values were greater than the results we obtained previously in later pregnancy (the AUCs of DD for GH, PE, and SPE were 0.560, 0.614, and 0.640, respectively) [26], but was slightly lower than the results in the second trimester (the AUCs of DD for GH, PE, and SPE were 0.642, 0.767, and 0.905, respectively) [27]. These findings indicated that the levels of DD in the first trimester had a higher diagnostic value than in the middle and late trimesters for GH and PE. However, for SPE, the diagnostic value of DD in the second trimester (AUC: 0.905) was slightly greater than that in the first trimester (AUC: 0.893). In summary, first trimester DD exhibited some value in predicting HDPs, although this index exhibited different diagnostic values for different gestational hypertensive diseases. If DD can be used to screen for HDPs in early pregnancy, then adverse pregnancy outcomes may be significantly improved. In addition, first trimester levels of PAPP-A and free *β*-hCG are often performed during prenatal screening for chromosomal aneuploidy abnormalities. If coupled with DD to screen for HDP, this combined strategy could be offered to pregnant women to predict the subsequent onset of HDP without the addition of invasive tests; this would also be more economical. Therefore, a low dose of aspirin may be offered at an early stage (before 16 weeks) to prevent HDPs [28].

Our study also showed that the levels of PAPP-A and free *β*-hCG in GH, PE, and SPE groups were lower than those in the control group, and showed statistical differences, while the MoM values of PAPP-A and free *β*-hCG in the GH, PE, and SPE groups were all lower than those in the control group; there were no significant differences with this regard. The reason for the discrepancy between the levels and MoM values of these indices may be that PAPP-A and free *β*-hCG levels were calibrated by gestational age and body weight. In addition, the results of a previous retrospective and case-control study, using on a larger dataset (902 cases) by our group, showed that the MoMs of PAPP-A and free *β*-hCG were significantly lower in the GH, PE, and SPE groups than in the control group; statistical differences were also evident [10]. These previous findings were not consistent with our present results, which may be related to the small sample size of this study. Thus, our findings must be validated in a larger dataset in future.

In the present study, maternal weight was significantly higher in the GH, PE, and SPE groups when compared with the control group, with statistically significant differences between groups (*P* < 0.001); thus, indicating that pregnant women who were overweight in early pregnancy were more likely to subsequently develop GH, PE, and SPE. Lewandowska et al. [29, 30] showed that compared to other risk factors (prior PE, pregnancy weight gain (GWG), infertility treatment, inter pregnancy interval, family history, the lack of vitamin supplementation, urogenital infection, and socioeconomic factors), the pre-pregnancy body mass index was the factor most likely to increase the likelihood of gestational hypertension and gestational diabetes mellitus. Therefore, the pre-pregnancy control of BMI is likely to be an effective means of preventing hypertensive disorders of pregnancy.

## Figures and Tables

**Table 1:** Comparison of demographic data in the first trimester between the different groups.

Groups	*n*	Maternal age (years)	Maternal weight (kg)	Gestational age at testing (days)
Control	150	28.23 (23.11-34.61)	51.00 (43.00-65.23)	91.00 (81.00-97.00)
GH	126	29.26 (21.46-34.48)	59.55 (44.51-91.30)	89.00 (80.00-97.00)
PE	53	29.26 (22.05-34.17)	57.00 (37.67-95.36)	90.00 (81.00-97.00)
SPE	41	28.52 (23.20-35.10)	56.40 (40.15-74.28)	90.00 (76.00-97.00)
*χ* ^2^		3.676	70.325	3.804
*P*		0.299	<0.001^∗^	0.283

GH: gestational hypertension; PE: preeclampsia; SPE: severe preeclampsia. ^∗^*P* < 0.001.

**Table 2:** DD, PAPP-A, free *β*-hCG, and NT concentration and MoM level in the different groups.

Groups	*n*	DD (ng/mL)	DD MoM	PAPP-A (mU/L)	PAPP-A MoM	Free *β*-hCG (ng/mL)	Free *β*-hCG MoM	NT (cm)	NT MoM
Control	150	226.60 (89.41-367.32)	0.84 (0.33-1.36)	4495 (1650-13545)	0.96 (0.36-2.75)	52.65 (16.87-156.23)	1.06 (0.40-2.88)	1.40 (0.73-2.34)	0.95 (0.52-1.53)
GH	126	269.52 (198.42-335.38)	1.00 (0.73-1.26)	3420 (837-10430)	0.96 (0.40-2.79)	42.4 (12.70-121.65)	0.97 (0.29-2.54)	1.40 (0.70-2.30)	0.96 (0.53-1.59)
PE	53	304.67 (193.88-361.89)	1.13 (0.72-1.34)	3720 (842-12120)	0.93 (0.30-2.20)	43.80 (10.61-315.00)	0.87 (0.29-6.93)	1.30 (0.56-2.71)	0.91 (0.40-1.69)
SPE	41	329.23 (256.03-397.95)	1.23 (0.95-1.64)	3320 (450-11495)	0.81 (0.14-2.74)	37.80 (15.10-168.05)	0.81 (0.35-2.63)	1.20 (0.70-1.95)	0.82 (0.52-1.19)
*χ* ^2^		99.804	70.325	18.282	3.917	12.983	5.510	3.784	8.475
*P*		<0.001^∗^	<0.001^∗^	<0.001^∗^	0.271	0.05^∗∗^	0.138	0.286	0.037^∗∗^

GH: gestational hypertension; PE: preeclampsia; SPE: severe preeclampsia; DD: D-dimer; PAPP-A: pregnancy-associated plasma protein A; free *β*-hCG: free beta human chorionic gonadotropin; NT: nuchal translucency; MoM: multiple of median. ^∗^*P* < 0.001; ^∗∗^*P* < 0.05.

**Table 3:** Predicting value of DD, PAPP-A, and free *β*-hCG in separate or joint screening for GH, PE, SPE, and HDP.

Screening indicators	Groups (*n*)	AUC	95% CI	*P*	Cut-off	Sensitivity	Specificity	Youden index
	GH (*n* = 126)							
DD		0.699	0.637-0.760	<0.001^∗^	0.725	0.992	0.353	0.345
PAPP-A		0.507	0.439-0.575	0.842	0.560	0.873	0.180	0.053
Free *β*-hCG		0.563	0.495-0.630	0.073	1.215	0.738	0.413	0.151
NT		0.512	0.440-0.585	0.736	0.825	0.730	0.336	0.066
DD+PAPP-A		0.700	0.639-0.760	<0.001^∗^	0.479	0.976	0.393	0.370
DD+free *β*-hCG		0.706	0.646-0.766	<0.001^∗^	0.551	0.960	0.393	0.354
PAPP-A+free *β*-hCG		0.582	0.515-0.649	0.019^∗∗^	1.047	0.667	0.520	0.187
DD+PAPP-A+free *β*-hCG		0.735	0.677-0.792	<0.001^∗^	1.750	0.659	0.700	0.359
PAPP-A+free *β*-hCG+NT		0.605	0.535-0.675	0.004^∗∗^	1.028	0.704	0.481	0.185
DD+PAPP-A+free *β*-hCG+NT		0.733	0.676-0.795	<0.001^∗^	0.461	0.965	0.420	0.385

	PE (*n* = 53)							
DD		0.784	0.718-0.850	<0.001^∗^	0.815	0.962	0.467	0.429
PAPP-A		0.510	0.420-0.600	0.830	0.725	0.774	0.313	0.087
Free *β*-hCG		0.585	0.497-0.673	0.067	1.370	0.868	0.340	0.208
NT		0.530	0.435-0.624	0.535	1.035	0.725	0.405	0.130
DD+PAPP-A		0.764	0.699-0.829	<0.001^∗^	0.474	0.925	0.513	0.438
DD+free *β*-hCG		0.773	0.709-0.838	<0.001^∗^	0.650	0.906	0.567	0.472
PAPP-A+free *β*-hCG		0.574	0.484-0.664	0.109	0.876	0.698	0.447	0.145
DD+PAPP-A+free *β*-hCG		0.795	0.734-0.857	<0.001^∗^	0.670	0.887	0.607	0.493
PAPP-A+free *β*-hCG+NT		0.595	0.503-0.686	0.048	1.153	0.431	0.740	0.172
DD+PAPP-A+free *β*-hCG+NT		0.808	0.745-0.871	<0.001^∗^	1.149	0.843	0.687	0.530

	SPE (*n* = 41)							
DD		0.893	0.848-0.939	<0.001^∗^	0.945	1.000	0.660	0.660
PAPP-A		0.592	0.488-0.696	0.071	0.955	0.683	0.500	0.183
Free *β*-hCG		0.572	0.474-0.671	0.156	1.015	0.659	0.533	0.192
NT		0.645	0.548-0.743	0.009^∗∗^	0.855	0.618	0.641	0.259
DD+PAPP-A		0.897	0.853-0.940	<0.001^∗^	0.615	0.951	0.727	0.678
DD+free *β*-hCG		0.874	0.825-0.922	<0.001^∗^	0.734	0.951	0.747	0.698
PAPP-A+free *β*-hCG		0.619	0.517-0.721	0.020^∗∗^	1.071	0.439	0.767	0.206
DD+PAPP-A+free *β*-hCG		0.904	0.862-0.946	<0.001^∗^	1.188	0.902	0.813	0.716
PAPP-A+free *β*-hCG+NT		0.733	0.642-0.825	<0.001^∗^	0.696	0.824	0.542	0.366
DD+PAPP-A+free *β*-hCG+NT		0.931	0.892-0.970	<0.001^∗^	1.634	0.912	0.878	0.790

	HDP (*n* = 220)							
DD		0.755	0.704-0.807	<0.001^∗^	0.915	0.755	0.640	0.395
PAPP-A		0.511	0.451-0.571	0.725	1.355	0.782	0.267	0.048
Free *β*-hCG		0.570	0.510-0.630	0.023^∗∗^	1.160	0.691	0.453	0.144
NT		0.525	0.461-0.589	0.440	1.035	0.675	0.405	0.080
DD+PAPP-A		0.711	0.656-0.767	<0.001^∗^	0.354	0.982	0.367	0.348
DD+free *β*-hCG		0.719	0.664-0.774	<0.001^∗^	0.372	0.982	0.367	0.348
PAPP-A+free *β*-hCG		0.558	0.489-0.618	0.056	0.887	0.823	0.280	0.103
DD+PAPP-A+free *β*-hCG		0.756	0.704-0.807	<0.001^∗^	1.177	0.809	0.600	0.409
PAPP-A+free *β*-hCG+NT		0.587	0.525-0.649	0.007^∗∗^	0.853	0.830	0.321	0.151
DD+PAPP-A+free *β*-hCG+NT		0.753	0.698-0.808	<0.001^∗^	0.865	0.855	0.542	0.397

DD: D-dimer; PAPP-A: pregnancy-associated plasma protein A; free *β*-hCG: free beta human chorionic gonadotropin; NT: nuchal translucency; MoM: multiple of the median; GH: gestational hypertension; PE: preeclampsia; SPE: severe preeclampsia; HDP: hypertensive disorders of pregnancy; AUC: area under the curve; LR: likelihood ratio. ^∗^*P* < 0.001; ^∗∗^*P* < 0.05.

**Table 4:** The value evaluation for the risk models.

Screening indicators	Groups (*n*)	FPR	FNR	PPV	NPV	+LR	-LR
	GH (*n* = 126)						
DD		0.647	0.008	0.563	0.981	1.534	0.022
PAPP-A		0.820	0.127	0.472	0.628	1.065	0.705
Free *β*-hCG		0.587	0.262	0.514	0.653	1.258	0.634
NT		0.664	0.270	0.491	0.587	1.100	0.803
DD+PAPP-A		0.607	0.024	0.575	0.952	1.609	0.061
DD+free *β*-hCG		0.607	0.040	0.571	0.922	1.583	0.101
PAPP-A+free *β*-hCG		0.480	0.333	0.538	0.650	1.389	0.641
DD+PAPP-A+free *β*-hCG		0.300	0.341	0.648	0.709	2.196	0.488
PAPP-A+free *β*-hCG+NT		0.519	0.296	0.544	0.649	1.357	0.615
DD+PAPP-A+free *β*-hCG+NT		0.580	0.035	0.594	0.932	1.664	0.083
	PE (*n* = 53)						
DD		0.533	0.038	0.389	0.972	1.804	0.081
PAPP-A		0.687	0.226	0.285	0.797	1.127	0.723
Free *β*-hCG		0.660	0.132	0.317	0.879	1.315	0.388
NT		0.595	0.275	0.322	0.791	1.218	0.679
DD+PAPP-A		0.487	0.075	0.402	0.951	1.900	0.147
DD+free *β*-hCG		0.433	0.094	0.425	0.944	2.090	0.166
PAPP-A+free *β*-hCG		0.553	0.302	0.308	0.807	1.262	0.676
DD+PAPP-A+free *β*-hCG		0.393	0.113	0.443	0.938	2.255	0.187
PAPP-A+free *β*-hCG+NT		0.260	0.569	0.393	0.770	1.662	0.768
DD+PAPP-A+free *β*-hCG+NT		0.313	0.157	0.512	0.918	2.694	0.228
	SPE (*n* = 41)						
DD		0.340	0	0.446	1.000	2.941	0
PAPP-A		0.500	0.317	0.272	0.852	1.366	0.634
Free *β*-hCG		0.467	0.341	0.278	0.851	1.411	0.640
NT		0.359	0.382	0.309	0.866	1.722	0.596
DD+PAPP-A		0.273	0.049	0.487	0.982	3.480	0.067
DD+free *β*-hCG		0.253	0.049	0.506	0.982	3.755	0.065
PAPP-A+free *β*-hCG		0.233	0.561	0.340	0.833	1.882	0.732
DD+PAPP-A+free *β*-hCG		0.187	0.098	0.569	0.968	4.834	0.120
PAPP-A+free *β*-hCG+NT		0.458	0.176	0.318	0.922	1.798	0.326
DD+PAPP-A+free *β*-hCG+NT		0.122	0.088	0.660	0.975	7.465	0.101
	HDP (*n* = 220)						
DD		0.360	0.245	0.755	0.640	2.096	0.384
PAPP-A		0.733	0.218	0.610	0.455	1.066	0.818
Free *β*-hCG		0.547	0.309	0.650	0.500	1.264	0.682
NT		0.595	0.325	0.634	0.449	1.134	0.803
DD+PAPP-A		0.633	0.018	0.695	0.932	1.550	0.050
DD+free *β*-hCG		0.633	0.018	0.695	0.932	1.550	0.050
PAPP-A+free *β*-hCG		0.720	0.177	0.626	0.519	1.143	0.633
DD+PAPP-A+free *β*-hCG		0.400	0.191	0.748	0.682	2.023	0.318
PAPP-A+free *β*-hCG+NT		0.679	0.170	0.651	0.553	1.222	0.530
DD+PAPP-A+free *β*-hCG+NT		0.458	0.145	0.740	0.710	1.867	0.268

DD: D-dimer; PAPP-A: pregnancy-associated plasma protein A; free *β*-hCG: free beta human chorionic gonadotropin; NT: nuchal translucency; MoM: multiple of the median; GH: gestational hypertension; PE: preeclampsia; SPE: severe preeclampsia; HDP: hypertensive disorders of pregnancy; AUC: area under curve; FNR: false negative rate; FPR: false positive rate; PPV: positive predictive value; NPV: negative predictive value; +LR: positive likelihood ratio; -LR: negative likelihood ratio.

## Data Availability

The datasets used and/or analyzed during the current study are available from the corresponding author on reasonable request.
